# DKA-Induced Takotsubo Cardiomyopathy in Patient with Known HOCM

**DOI:** 10.1155/2017/4287125

**Published:** 2017-04-03

**Authors:** Ayla Gordon, Gina LaCapra, Roberto Roberti

**Affiliations:** Overlook Medical Center, Summit, NJ, USA

## Abstract

The first published case of Diabetic Ketoacidosis-induced Takotsubo cardiomyopathy was in 2009. Our patient is the 1st reported case of Diabetic Ketoacidosis- (DKA-) induced Takotsubo cardiomyopathy (TC) in a patient with known hypertrophic cardiomyopathy (HOCM) in the United States. In the literature, there are only two examples linking DKA to TC; however, this report focuses on the biochemical and physiological causes of TC in a patient with known HOCM and new-onset DKA. TC in previously diagnosed HOCM poses particular complications. With the above patient's baseline outflow tract obstruction due to septal hypertrophy, the acute reduction in EF due to TC resulted in transient drop in brain perfusion and, therefore, syncope.

## 1. Introduction

The most common documented cause of Takotsubo cardiomyopathy (TC) is a neurohormonal release of catecholamines. However, in this case report of a man presenting with syncope, we describe DKA as the physiological stressor leading to TC. Upon workup for this patient's loss of consciousness, initial labs revealed a metabolic acidotic state and a glucose level of 526 mg/dL. Troponin level was 13.8 ng/mL with EKG showing ST elevations in leads V3–V6. Emergent coronary angiography demonstrated normal coronaries with a clinical picture suggesting TC. Transthoracic echo confirmed TC. DKA was controlled by day 4 of hospital stay and repeat echo on day 5 showed documented resolution of apical ballooning. The correlation is explained physiologically; DKA increases serum catecholamines, and the metabolic acidosis that ensues prevents the healthy myocyte's stepwise chemical channel processes, most notably the sarcoplasmic reticulum's ability to release Ca++.

Typical treatment of TC is supportive in nature, and typical resolution is seen within 2 months. However, when there is a physiological underlying cause, the aim is to treat the cause first. In this case, DKA-induced physiological stress on the myocardium leads to apical stunning. When DKA was treated and glucose levels were brought within normal range, repeat echo revealed an improved EF and normal ventricular motion and, therefore, overall resolution of TC.

## 2. Case

The patient is a 66-year-old male with a history of hypertension, hypertrophic obstructive cardiomyopathy, Meniere's disease with intermittent lightheadedness, and gait instability that is controlled with intermittent prednisone. His usual symptoms progressively worsened with new polyuria, polydipsia, decreased oral intake, and nausea. On the day of admission, he complained of lightheadedness as he got up from the toilet and lost consciousness. He had never had a prior syncopal episode.

**Figure 1:**
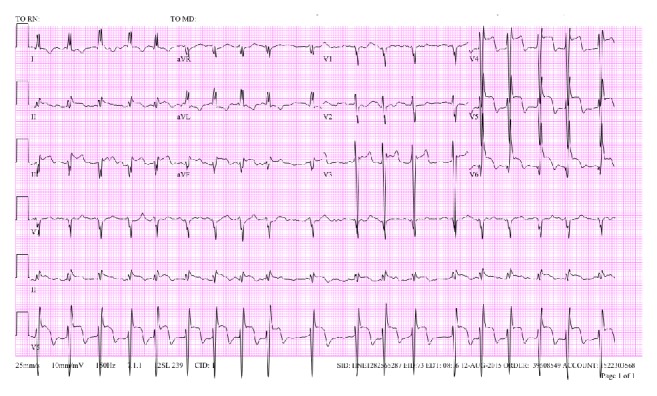
EKG showed atrial fibrillation with rapid ventricular response as well as ST elevations in leads II, III, aVF, and V3–V6.

Initial physical exam revealed irregularly irregular heart rate at 116 bpm, respiratory rate 26, and BP 108/74 with negative orthostatics. Initial labs revealed glucose of 526 mg/dL and blood gas pH of 7.12. Troponin was 13.8 ng/mL. HgbA1c of 11.8 later revealed a new diagnosis of diabetes mellitus. EKG showed atrial fibrillation with rapid ventricular response as well as ST elevations in leads II, III, aVF, and V3–V6 (Figure 1).

## 3. Hospital Course

**Figure 2:**
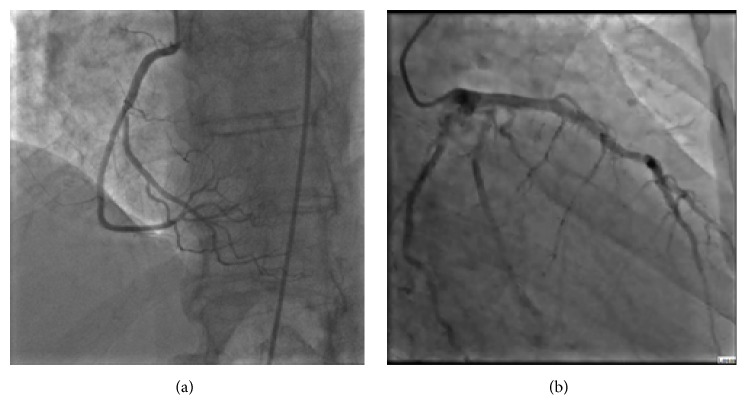
(a) and (b): cardiac catheterization on day of admission revealing no obstructive coronary artery disease in RCA and LAD, respectively.

**Figure 3:**
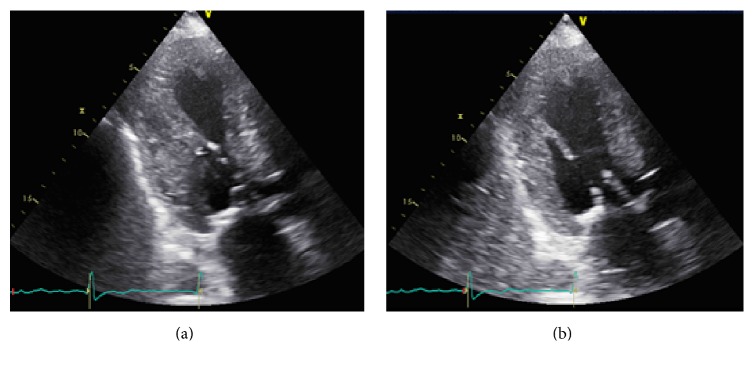
(a) and (b): echocardiogram on day of admission showing the apical ballooning typical of TC in systole and diastole, respectively.

**Figure 4:**
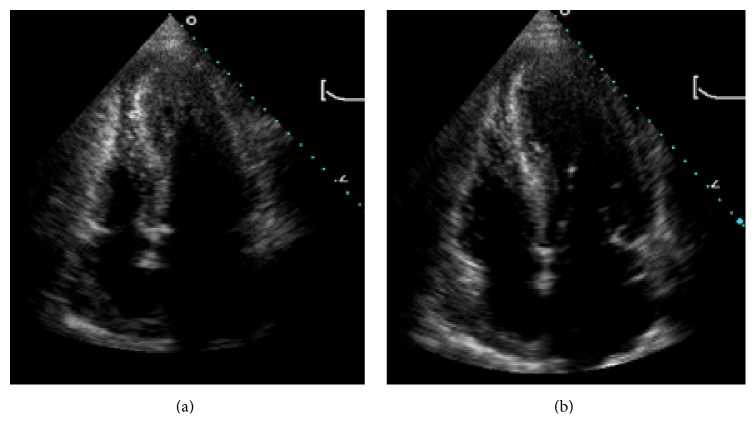
(a) and (b): patient's baseline echo showing asymmetric septal hypertrophy in systole and diastole, respectively.

Patient was admitted with ST elevation MI. Emergent coronary angiography demonstrated normal coronaries (Figures 2(a) and 2(b)) with anterolateral, apical, and inferoapical dyskinesis and basal anterior and basal inferior wall hyperkinesis; ejection fraction (EF) was 20%. Clinical picture suggested TC. Transthoracic echocardiography also revealed apical hypokinesis; EF was 35–40%. Findings were consistent with TC (Figures 3(a) and 3(b)). When examining an old echocardiogram, patient had documented asymmetric septal hypertrophy, systolic anterior motion of mitral valve, and an intraventricular gradient of 32 mmhg with an EF of 75% (Figures 4(a) and 4(b)). The baseline echo showed typical characteristics of HOCM yet supported that this presentation of heart failure is a new and acute finding.

**Figure 5:**
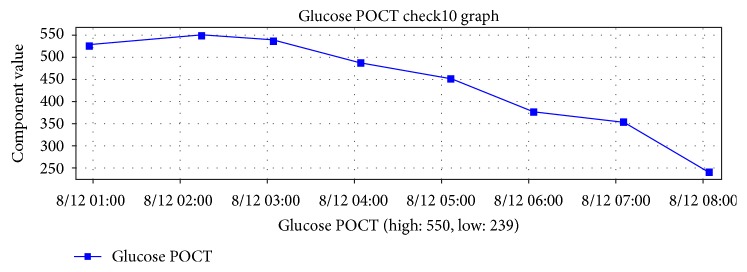
Glucose level since diagnosis of DKA.

The patient was treated with an insulin drip and was started on anticoagulation. His atrial fibrillation was rate controlled with carvedilol and cardizem. Due to Diabetic Ketoacidosis (DKA) in setting of new and acute cardiomyopathy, moderate hydration was started to avoid risk of pulmonary overload. DKA was controlled by day 4 of hospital stay (Figure 5) and repeat echo on day 5 showed documented resolution of apical ballooning. EF improved to 45%. The resolution of TC with DKA treatment, seen as improvement ejection fraction and left ventricular motion improvement on echocardiogram, suggests a direct association between the two conditions.

## 4. Discussion

TC is a reversible heart failure with characteristic left ventricular dysfunction in the setting of ST elevations yet absence of coronary artery disease. There have been 4 classifications reported in Lithuania: Takotsubo type, reverse Takotsubo type, mid-ventricular type, and localized type [1]. In a large cohort study out of Switzerland, over 13,000 angiographies were performed with a 1.7% incidence of Takotsubo in patients presenting with acute coronary syndromes; 85% of them were women [2].

TC is believed to be caused by overactivity of the sympathetic system. The excess stress-induced catecholamines have the greatest effect on the cardiac apex due to its high concentration of beta-adrenoreceptors. This causes hyperstimulation of the catecholamine receptors and an inability to contract (“stunned” effect), seen as hypokinesis of the apex typically described as “apical ballooning.” The most common documented cause of this type or cardiomyopathy is emotional neurohormonal stress; however, it has also been reported secondary to physiological stressors, as seen above. In this case report, we describe an association of TC with DKA being the physiological stressor.

During DKA, there is a physiological increase in other hormones such as growth hormone, cortisol, glucagon, and catecholamines which all play a part in worsening hyperglycemia [3]. In 2009, the first case of DKA-induced TC was published [4]. Our patient is the 1st reported case of DKA-induced TC in a patient with known HOCM in the United States.

In our patient, not only does DKA increase serum catecholamines, but the acidosis also contributes to the dysfunction. During DKA, hepatic oxidation of free fatty acids produces an increased amount of ketones (most commonly acetoacetate and hydroxybutyrate). Serum ketones are cardioprotective by supplying heart muscle with an energy substrate other than free fatty acids. However, when ketone concentrations become exceedingly high within the blood, the patient enters an acidotic state preventing the healthy stepwise myocyte chemical channel processes. The most affected function is the sarcoplasmic reticulum's ability to release Ca++. The ultimate effect is inhibition of normal myocyte contractility. This theory is supported by the pathological findings of sarcolipin within left ventricular myocytes during the acute phase of Takotsubo, which interrupt regulation of intracellular calcium [5].

There is a case report of a patient who had both DKA and hypothermia who developed TC [6]. In this case, hypothermia is described as the main cause of catecholamine release while coupled with an acidotic state, both causing hyperactivity of the ventricular bases and, therefore, TC. This is yet another case, where the combination of increased serum catecholamines and acidosis may result in TC.

Typical treatment of TC is supportive in nature and typical resolution is seen within 2 months [7]. However, when there is a physiological underlying cause, the aim is to treat the cause first. In this case, DKA-induced physiological stress on the myocardium leads to apical stunning. When DKA was treated and glucose levels were brought within normal range, repeat echo revealed an improved EF and normal ventricular motion and, therefore, overall resolution of TC.

TC in previously diagnosed HOCM poses particular complications. With the above patient's baseline outflow tract obstruction due to septal hypertrophy of approximately 3.2 cm, the acute presentation of dynamic LV outflow tract obstruction and reduced EF due to TC resulted in transient drop in brain perfusion and therefore syncope. Typical symptomatic presentation of TC mimics acute coronary syndromes and is usually only diagnosed after direct visualization of the apical ballooning. However, the patient's history of HOCM and new acute cardiomyopathy put him at an increased risk of cardiac shock. Due to quick reversibility of DKA-induced TC with rapid glucose control, the patient's cardiac function was able to return to baseline.
